# Supplementary figures and images for: Drosophila insulin-like peptide 2 mediates dietary regulation of sleep intensity
Source: PLoS Genet. 2020 Mar 11;16(3):e1008270. doi: 10.1371/journal.pgen.1008270 (PMC7089559; doi:10.1371/journal.pgen.1008270)

**A**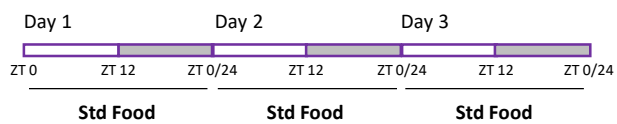**B**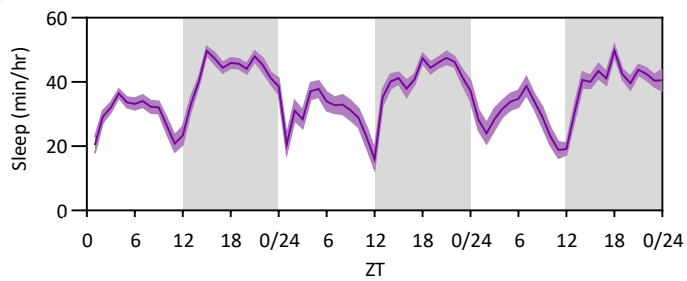**C**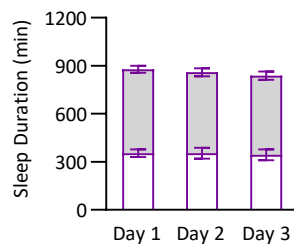**D**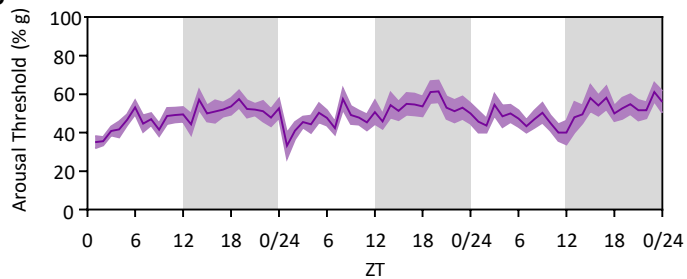**E**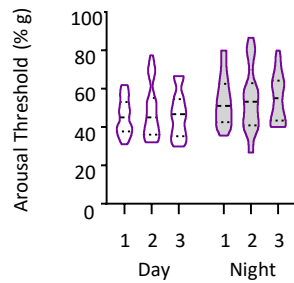

Supplement: S1 Fig — (A) Total sleep and arousal threshold were assessed on standard food over a 3-day period. (B) Sleep profile. (C) There is no change in sleep duration over a 3-day period (two-way ANOVA: F2,168 = 0.24, P<0.79). This is consistent during the day (ANOVA: F2,84 = 0.03, P<0.96) and night (ANOVA: F2,84 = 0.35, P<0.70). (D) Profile of arousal threshold. (E) There is no change in arousal threshold over a 3-day period (REML: F2,56 = 0.17, P<0.83). This is consistent during the day (Kruskal-Wallis test: H = 0.09, P<0.95; N = 29) and the night (Kruskal-Wallis test: H = 0.23, P<0.89; N = 29). For profiles, shaded regions indicate +/- standard error from the mean. White background indicates daytime, while gray background indicates nighttime. For sleep measurements, error bars represent +/- standard error from the mean. For arousal threshold measurements, the median (dashed line) as well as 25th and 75th percentiles (dotted lines) are shown. (PDF) [file pgen.1008270.s001.pdf]

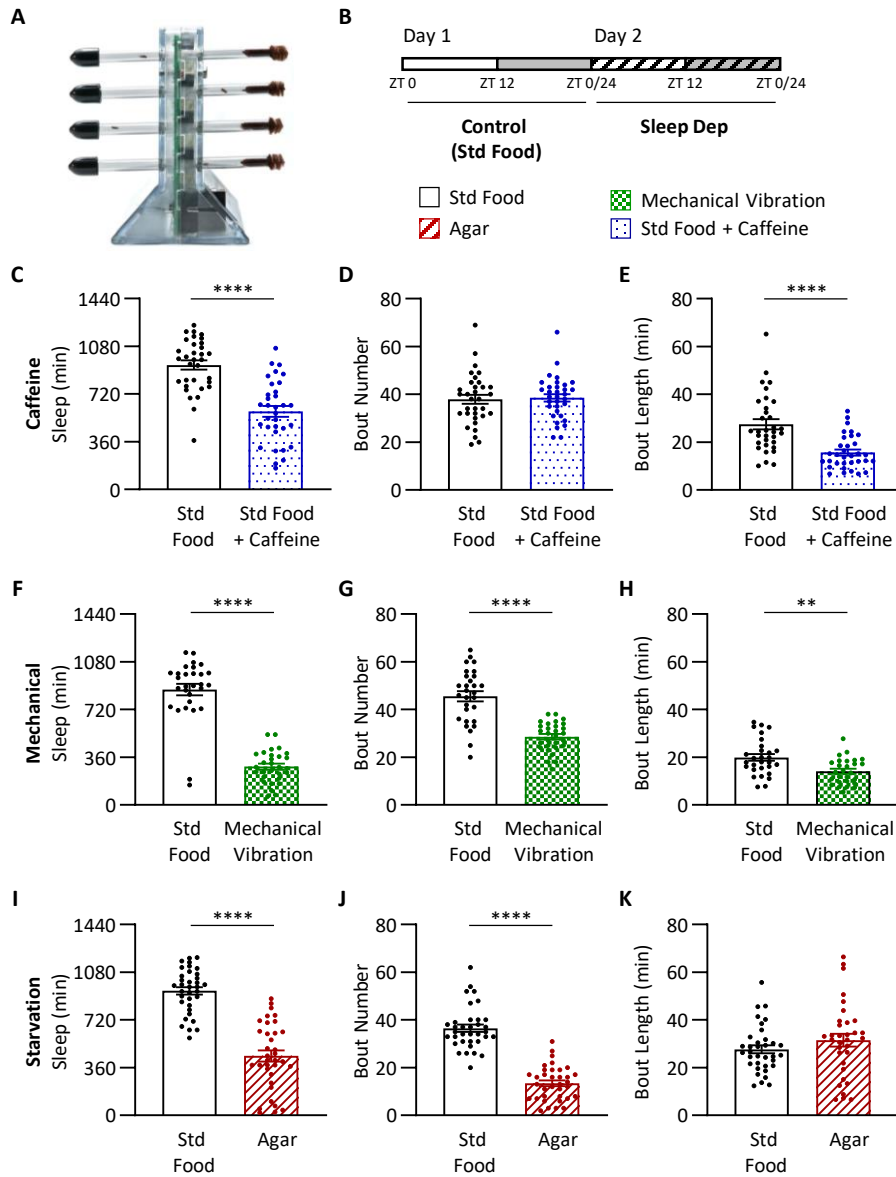

Supplement: S2 Fig — (A) Sleep traits were measured using the Drosophila Activity Monitoring (DAM) system. Individual flies were placed inside plastic tubes containing standard food at one end and a foam plug at the other. Flies were then allowed to acclimate for 24 hrs. (B) At ZT0, baseline sleep was measured on a standard food diet (Day 1). At ZT0 of the following day, flies were sleep deprived using 0.5mg/mL caffeine, mechanical vibration, or starved for 24 hrs. (C) Total sleep duration significantly decreases when caffeine is added to a standard food diet (t-test: t64 = 6.54, P<0.0001). (D,E) This is not due to a change in bout number (t-test: t64 = 0.24, P<0.80), but rather a significant decrease in bout length (t-test: t64 = 4.82, P<0.0001). (F) Total sleep duration significantly decreases when flies are mechanically sleep deprived (t-test: t56 = 12.06, P<0.0001). (G,H) This is caused by a significant decrease in bout number (t-test: t56 = 7.13, P<0.0001) as well as a significant decrease in bout length (t-test: t54 = 3.24, P<0.0020). (I) Total sleep duration significantly decreases during starvation (t-test: t68 = 9.69, P<0.0001). (J,K) This is due to a significant decrease in bout number (t-test: t68 = 11.85, P<0.0001), but not bout length (t-test: t68 = 1.22, P<0.22). Error bars represent +/- standard error from the mean. ** = P<0.01; **** = P<0.0001. (PDF) [file pgen.1008270.s002.pdf]

**A** *w<sup>1118</sup>* Males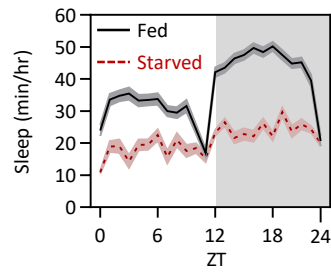**B**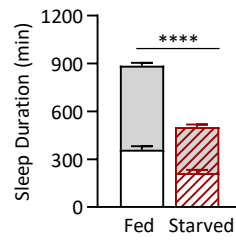**C**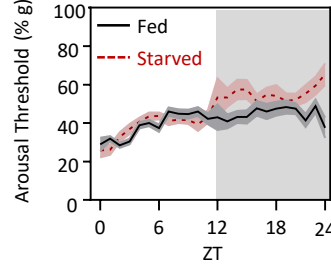**D**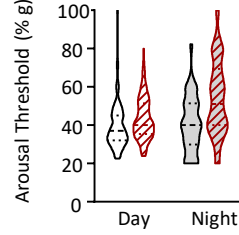**E** Canton-S Females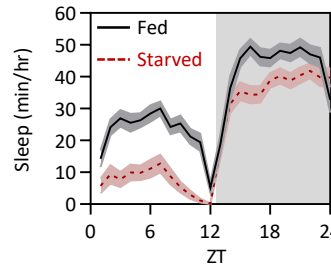**F**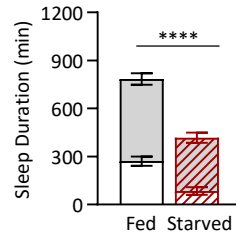**G**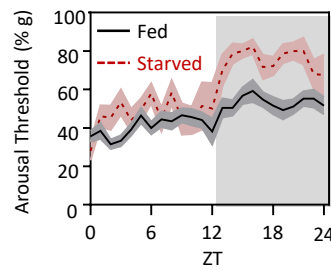**H**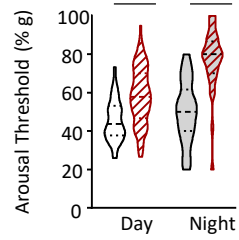

Supplement: S3 Fig — All experiments were performed as described in Fig 2A. (A-D) Sleep and arousal threshold measurements in w1118 male flies. (A) Sleep profiles of fed and starved flies. (B) Sleep duration decreases in the starved state (two-way ANOVA: F1,152 = 101.4, P<0.0001), and occurs during both the day (P<0.0001) and night (P<0.0001). (C) Profile of arousal threshold of fed and starved flies. (D) Arousal threshold significantly increases in the starved state (REML: F1,71 = 40.81, P<0.0001), but occurs only at night (day: P<0.11; night: P<0.0001). (E-H) Sleep and arousal threshold measurements in female Canton-S flies. (E) Sleep profiles of fed and starved flies. (F) Sleep duration decreases in the starved state (two-way ANOVA: F1,122 = 36.92, P<0.0001), and occurs during both the day (P<0.0001) and night (P<0.0001). (G) Profile of arousal threshold of fed and starved flies. (H) Arousal threshold significantly increases in the starved state (REML: F1,52 = 62.11, P<0.0001), and occurs both during the day and at night (day: P<0.0008; night: P<0.0001). For profiles, shaded regions indicate +/- standard error from the mean. White background indicates daytime, while gray background indicates nighttime. For sleep measurements, error bars represent +/- standard error from the mean. For arousal threshold measurements, the median (dashed line) as well as 25th and 75th percentiles (dotted lines) are shown. *** = P<0.001; **** = P<0.0001. (PDF) [file pgen.1008270.s003.pdf]

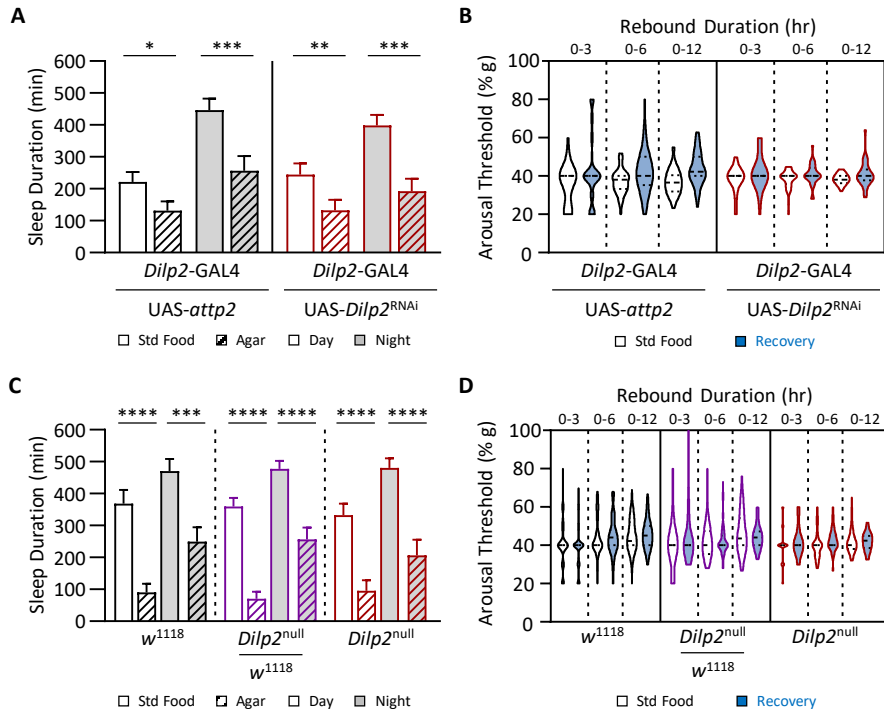

Supplement: S4 Fig — (A) Compared to the control, knockdown of Dilp2 in Dilp2-expressing does not affect sleep duration (two-way ANOVA: F1,146 = 2.16, P<0.14), and both genotypes suppressed sleep during starvation (two-way ANOVA: F1,146 = 26.78, P<0.0001). For each genotype, post hoc analyses revealed a significant decrease in sleep duration when starved both during the day (Dilp2-GAL4>UAS-attp2: P<0.03; Dilp2-GAL4>UAS-Dilp2RNAi: P<0.0048) and night (Dilp2-GAL4>UAS-attp2: P<0.0008; Dilp2-GAL4>UAS-Dilp2RNAi: P<0.0002). (B) Compared to the control, knockdown of Dilp2 in Dilp2-expressing neurons has no effect on arousal threshold during recovery (REML: F1,76 = 0.46, P<0.49). (C) In comparison to the control, there is no effect on sleep duration in heterozygotes or Dilp2null flies (two-way ANOVA: F1,231 = 0.19, P<0.81), and all genotypes suppressed sleep during starvation (two-way ANOVA: F1,231 = 59.11, P<0.0001). For all three genotypes, post hoc analyses revealed a significant decrease in sleep duration when starved both during the day (w1118: P<0.0001; w1118/Dilp2null: P<0.0001; Dilp2null: P<0.0001) and night (w1118: P<0.0002; w1118/Dilp2null: P<0.0001; Dilp2null: P<0.0001). (D) In comparison to the control, there is no effect on arousal threshold during recovery in heterozygotes or Dilp2null flies (REML: F2,117 = 1.42, P<0.23). For sleep measurements, error bars represent +/- standard error from the mean. For arousal threshold measurements, the median (dashed line) as well as 25th and 75th percentiles (dotted lines) are shown. Measurements of homeostatic rebound were assessed in 3-, 6-, and 12-hr increments. * = P<0.05; ** = P<0.01; *** = P<0.001; **** = P<0.0001. (PDF) [file pgen.1008270.s004.pdf]

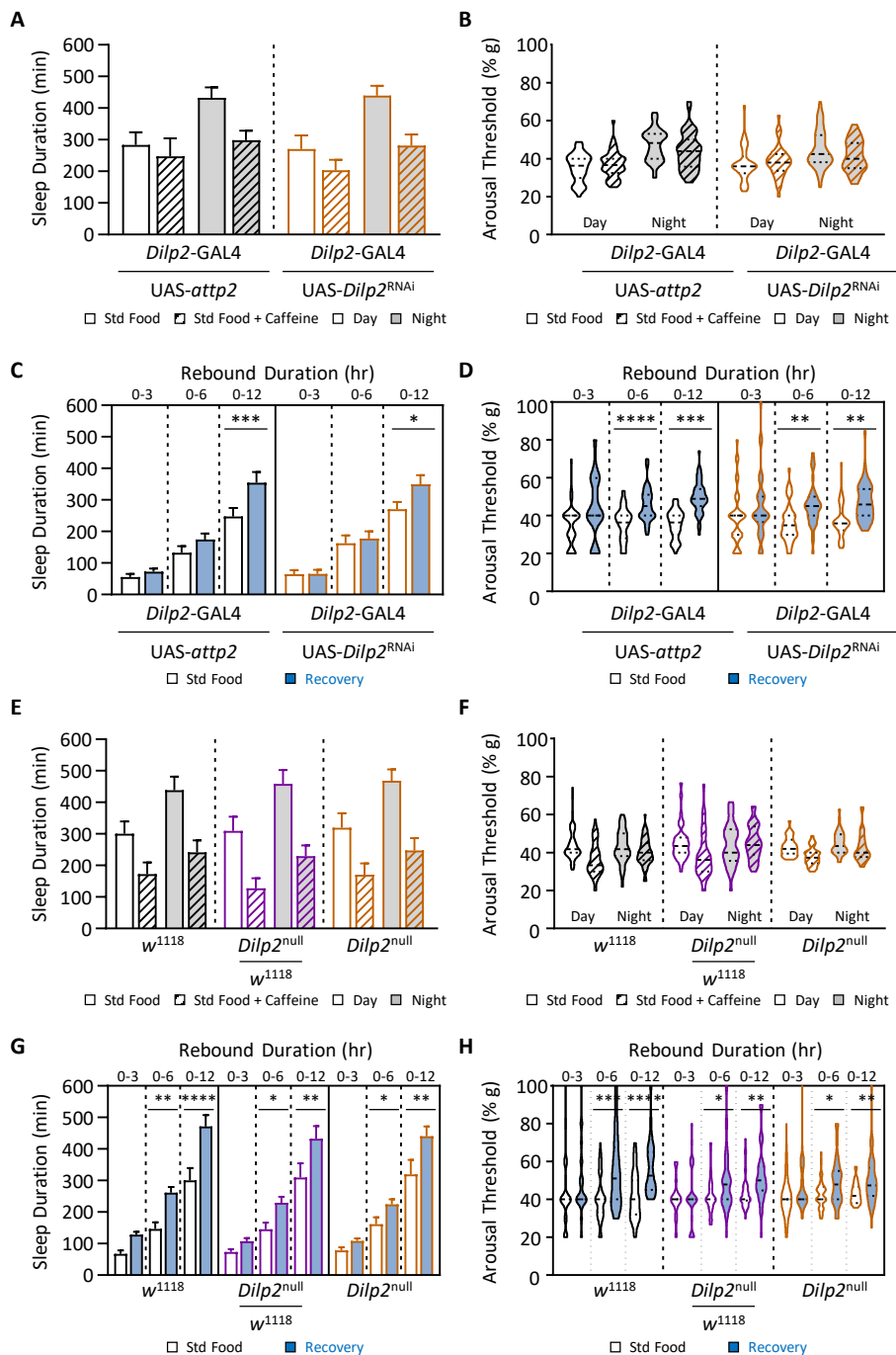

Supplement: S5 Fig — Total sleep and arousal threshold during sleep deprivation and recovery were assessed as described in Fig 1A. Flies were sleep deprived by adding 0.5mg/mL caffeine to their diet. (A) There is no effect of genotype on nighttime sleep duration (two-way ANOVA: F1,154 = 0.02, P<0.87), and all genotypes suppressed sleep when fed caffeine (two-way ANOVA: F1,154 = 20.56, P<0.0001). Post hoc analyses revealed a significant decrease in nighttime sleep duration when fed caffeine for both genotypes (Dilp2-GAL4>UAS-attp2: P<0.0069; Dilp2-GAL4>UAS-Dilp2RNAi: P<0.0015). (B) Similar to the control, knockdown of Dilp2 in Dilp2-expressing neurons has no effect on nighttime arousal threshold when fed caffeine (REML: F1,78 = 2.22, P<0.13). (C) There is no effect of genotype on sleep duration (two-way ANOVA: F1,154 = 0.13, P<0.71), however there was a significant effect of recovery (two-way ANOVA: F1,154 = 15.80, P<0.0001). For both genotypes, post hoc analyses revealed a significant increase in sleep duration after 6 hrs of recovery (Dilp2-GAL4>UAS-attp2: 0–3 hrs: P<0.90; 0–6 hrs: P<0.34; 0–12 hrs: P<0.0005; Dilp2-GAL4>UAS-Dilp2RNAi: 0–3 hrs: P<0.99; 0–6 hrs: P<0.94; 0–12 hrs: P<0.03). (D) There was no effect of genotype on arousal threshold (REML: F1,78 = 0.06, P<0.79), however there was a significant effect of recovery (REML: F1,76 = 106.1, P<0.0001). For all both genotypes, post hoc analyses revealed a significant increase in arousal threshold after 3 hrs of recovery (Dilp2-GAL4>UAS-attp2: 0–3 hrs: P<0.05; 0–6 hrs: P<0.0001; 0–12 hrs: P<0.0001; Dilp2-GAL4>UAS-Dilp2RNAi: 0–3 hrs: P<0.09; 0–6 hrs: P<0.0064; 0–12 hrs: P<0.0012). (E) There was no effect of genotype on nighttime sleep duration (two-way ANOVA: F2,208 = 0.11, P<0.88), and all genotypes suppressed sleep when fed caffeine (two-way ANOVA: F2,208 = 45.32, P<0.0001). Post hoc analyses revealed a significant decrease in nighttime sleep duration when fed caffeine for all three genotypes (w1118: P<0.0016; w1118/Dilp2null: P<0.000 [file pgen.1008270.s005.pdf]

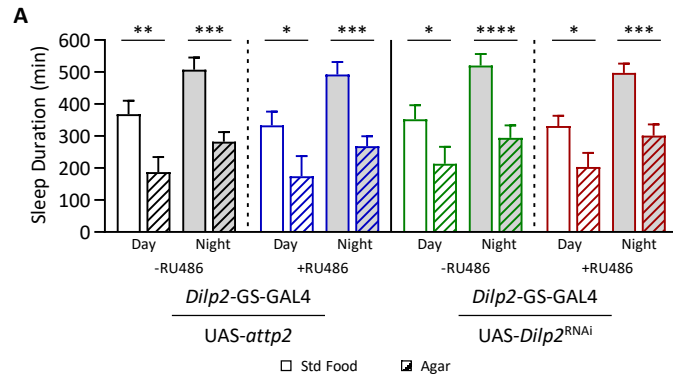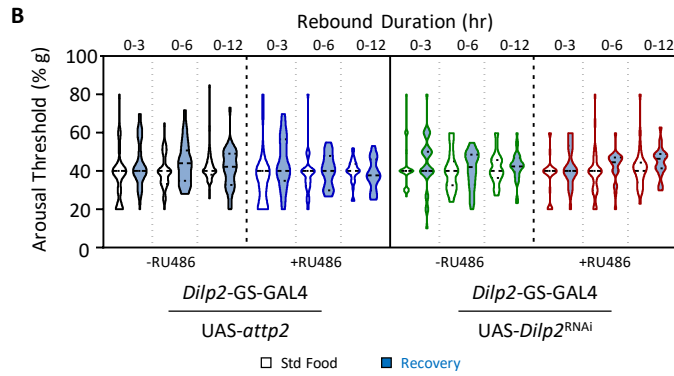

Supplement: S6 Fig — (A) There is no effect of genotype on nighttime sleep duration (two-way ANOVA: F3,67 = 0.19, P<0.89), and all genotypes suppressed sleep during starvation (two-way ANOVA: F1,67 = 74.34, P<0.0001). Post hoc analyses revealed a significant decrease in sleep duration when starved both during the day (Dilp2-GS-GAL4>UAS-attp2/-RU486: P<0.0048; Dilp2-GS-GAL4>UAS-attp2/+RU486: P<0.04; Dilp2-GS-GAL4>UAS-Dilp2RNAi/-RU486: P<0.04; Dilp2-GS-GAL4>UAS-Dilp2RNAi/+RU486: P<0.02) and night (Dilp2-GS-GAL4>UAS-attp2/-RU486: P<0.0002; Dilp2-GS-GAL4>UAS-attp2/+RU486: P<0.0003; Dilp2-GS-GAL4>UAS-Dilp2RNAi/-RU486: P<0.0001; Dilp2-GS-GAL4>UAS-Dilp2RNAi/+RU486: P<0.006). (B) Compared to the controls, knockdown of Dilp2 in Dilp2-expressing neurons (Dilp2-GS-GAL4>UAS-Dilp2RNAi/+RU486) has no effect on arousal threshold following 24 hrs of starvation (REML: F3,134 = 1.48, P<0.22). For sleep measurements, error bars represent +/- standard error from the mean. For arousal threshold measurements, the median (dashed line) as well as 25th and 75th percentiles (dotted lines) are shown. Measurements of homeostatic rebound were assessed in 3-, 6-, and 12-hr increments. * = P<0.05; ** = P<0.01; *** = P<0.001; **** = P<0.0001. (PDF) [file pgen.1008270.s006.pdf]

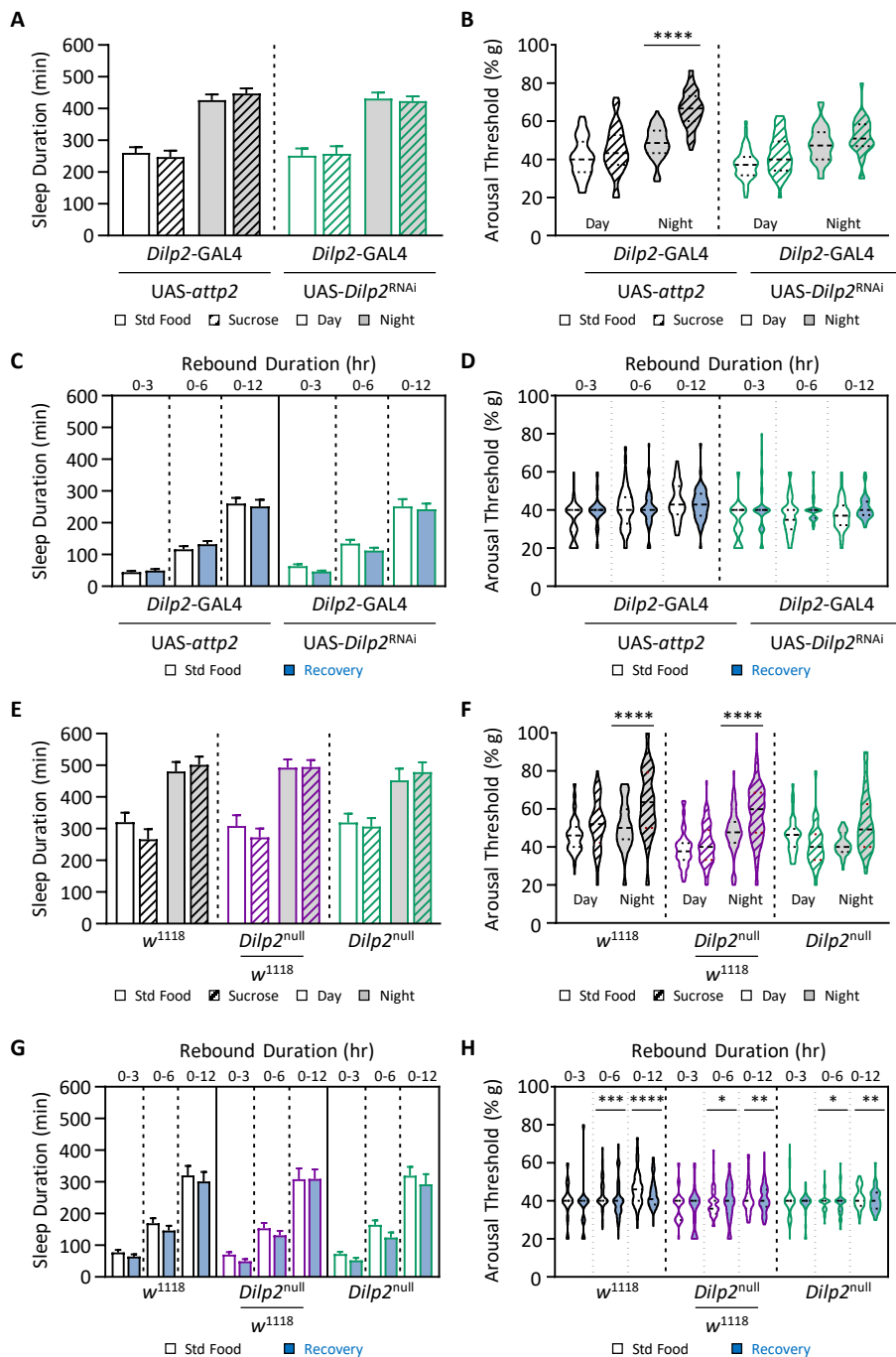

Supplement: S7 Fig — Total sleep and arousal threshold were assessed as described in Fig 4A. On Day 2 of testing, flies were fed a diet of 5% sucrose. (A) Compared to the control, knockdown of Dilp2 in Dilp2-expressing neurons has no effect on sleep duration when fed a sucrose-only diet (two-way ANOVA: F1,150 = 0.21, P<0.64). (B) There is a significant effect of genotype on nighttime arousal threshold (REML: F1,75 = 26.51, P<0.0001). Post hoc analyses revealed that while controls significantly increase nighttime arousal threshold when fed a sucrose-only diet (P<0.0001), there is no effect upon knockdown of Dilp2 in Dilp2-expressing neurons (P<0.13). (C) Similar to the control, knockdown of Dilp2 in Dilp2-expressing neurons does not change sleep duration during recovery when fed a sucrose-only diet (two-way ANOVA: F1,150 = 0.19, P<0.66). (D) There is a significant effect of genotype on arousal threshold during recovery (REML: F1,75 = 6.21, P<0.01). However, post hoc analyses revealed no differences in arousal threshold in the control (P<0.20), nor upon knockdown of Dilp2 in Dilp2-expressing neurons (P<0.12). (E) In comparison to the control, there is no effect on nighttime sleep duration in heterozygotes or Dilp2null flies when fed a sucrose-only diet (two-way ANOVA: F2,216 = 0.02, P<0.97). (F) There is a significant effect of genotype on nighttime arousal threshold (REML: F2,108 = 5.93, P<0.0032). Post hoc analyses revealed that while controls and heterozygotes significantly increase nighttime arousal threshold when fed a diet of sucrose (w1118: P<0.0001; w1118/Dilp2null: P<0.0001), there is no effect on arousal threshold in Dilp2null flies (P<0.10). (G) Similar to the control, there is no effect on sleep duration during recovery in heterozygotes or Dilp2null flies (two-way ANOVA: F2,216 = 0.01, P<0.98). (H) There is a significant effect of genotype on arousal threshold during recovery (REML: F2,108 = 14.58, P<0.0001). However, post hoc analyses revealed no differences in arousal thresh [file pgen.1008270.s007.pdf]
