# Supplementary material for: Drosophila insulin-like peptide 2 mediates dietary regulation of sleep intensity
Source: PLoS Genet. 2020 Mar 11;16(3):e1008270. doi: 10.1371/journal.pgen.1008270 (PMC7089559; doi:10.1371/journal.pgen.1008270)
Supplement: Attachment — Submitted filename: PLOS Genetics Brown et al 2019.pdf [file pgen.1008270.s009.pdf]

Understanding how sleep regulatory processes are influenced by dietary macronutrient availability and specific metabolic processes has garnered interest from both the neurobiology and metabolism community, especially for those who use genetic model organisms. Brown et al. adds to this growing trend by providing evidence demonstrating that dietary yeast and DILP2 signaling can modulate sleep intensity. Unlike other types of sleep deprivation paradigms which results in a significant increase in recovery sleep, the authors conclude that starvation promotes deeper sleep 12 hr after starvation onset, preventing subsequent sleep rebound. Examination of arousal threshold and metabolic respiration across different diets revealed loss of dietary yeast was responsible for the increased sleep depth, an intriguing observation that was accompanied by a reduction in metabolic rate. The authors finally identify a candidate signaling molecule, DILP2, that is required for the changes in sleep intensity during and rebound following starvation.

Overall, the findings and conclusions drawn are noteworthy and novel. However, I have several general issues with this manuscript that I believe must be addressed, including (i) possible inconsistencies between the starvation-induced sleep intensity and rebound phenotypes reported here and published previously by others; (ii) the lack of pursuit to identify the macronutrient responsible for the changes in sleep depth; and (iii) the minimal evidence supporting a role for DILP2 signaling in modulating sleep intensity and rebound following starvation, along with its connection to dietary yeast. If the authors address the following concerns, that would greatly strengthen my opinion of the manuscript and could merit publication in PLOS Genetics.

1. According to data presented in Figure 1, the authors conclude that starvation-induced sleep loss does not produce a subsequent increase in rebound sleep. However, Keene et al. previously reported that “male and female flies rebound in the 4 hr after food deprivation” (Keene et al., 2010), contradicting a major conclusion made in this manuscript. Can the authors comment on or resolve this discrepancy?
2. Regarding Figure 1, it is worth noting that the greatest differences in recovery sleep are observed within the first few hours immediately following an acute sleep deprivation protocol (Shaw et al., 2000; Hendricks et al., 2000; Huber et al., 2004; Keene et al., 2010; Dubowy et al., 2016; Vienne et al., 2016; Sonn et al., 2018). Since the authors quantified recovery sleep across a 12 hr interval, immediate changes in recovery sleep may have gone unnoticed. I recommend presenting sleep amount profiles illustrating the baseline, sleep deprivation, and post-deprivation recovery period alongside the 12 hr recovery violin plots. This would allow the authors to further demonstrate how sleep rebound does/does not change across time for each of the different sleep deprivation paradigms presented in Figure 1.
3. Have the authors looked at sleep fragmentation and architecture parameters during either the sleep deprivation period or 24 hr recovery period in an attempt to independently support the arousal threshold observations? For example, one would expect an increase in average sleep bout duration and a decrease in the number of sleep bouts to complement an increase in sleep depth; a trend that would be observed in 24 hr starved flies during the night period. Such an analysis would have to be done without the use of the DART system, since hourly disruption of sleep could alter interpretation of the fragmentation parameters.
4. The Materials and Methods section describes how arousal threshold was tested, yet never explains how this data was used to define and quantify arousal threshold throughout the manuscript. Exactly what value is being reported in the arousal threshold plots? Does it refer to the average amount of stimulus required to arouse a specified percentage of the sleeping population?
5. Even though the authors conclude that changes to sleep intensity and metabolic rate are primarily driven by dietary yeast availability, little is said or done to determine which macronutrient provided by dietary yeast is responsible for these changes. The evidence strongly points towards a role for

protein and/or amino acids in modulating sleep depth, which would be consistent with recent findings demonstrating the effects that specific amino acids have on sleep regulatory processes (Dai et al., 2019; Sonn et al., 2018; Ki and Lim, 2019). Could the authors (i) use a chemically defined food recipe devoid of carboyhydrates (e.g., Lee and Micchelli, 2013) in place of dietary yeast alone conditions, with the expectation that it phenocopies the dietary yeast alone response or (ii) by systematically adding back specific amino acids to a sugar-only diet in an attempt to revert the sleep intensity phenotype?

6. Flies fed a standard yeast-sugar diet containing the glycolysis inhibitor 2-deoxygluose caused a decrease in sleep duration and increase in arousal threshold, which is used to establish that "metabolic deprivation, rather than lack of sensory inputs, account for the changes in sleep…" (Figure S5). These results also imply that glucose metabolism is capable of modulating sleep intensity, despite the observation that environmental sugar availability does not drive changes in sleep depth. Can the authors comment on or resolve this possible conflict? Furthermore, could experiments be designed to assess the contribution of protein metabolism/homeostasis to changes in arousal threshold 12 hr after dietary change onset, which would better complement the data implicating the effects of dietary yeast on sleep intensity?
7. Given the widespread physiological behavioral changes that often accompany DILP2 loss throughout development, have the authors considered assessing whether acute loss of DILP2 in adults flies during the starvation period is enough to suppress sleep depth during starvation and promote sleep rebound post-starvation? The authors can either try (i) *DILP2-GeneSwitch-GAL4* driving *UAS-DILP2-RNAi* or (ii) [*DILP2-GAL4* + *tubGAL80^{ts}*] driving *UAS-DILP2-RNAi* to address this question.
8. Does acute overexpression of DILP2 in the IPCs (e.g., *DILP2-GeneSwitch-GAL4* driving *UAS-DILP*) cause an increase in sleep depth on standard diet or yeast-only? If it does, this experiment would further support DILP2 signaling involvement in modulating sleep depth.
9. Can the authors provide additional evidence that DILP2 signaling and/or DILP2 IPC activity is enhanced in response to their starvation or sucrose-only dietary conditions? While the Discussion section mentions "there are conflicting reports on whether *Dilp2* expression is modulated during starvation", I'm hoping the authors can provide clarity to this situation in this manuscript, which would further strengthen the case that increased DILP2 signaling is indeed responsible for the sleep intensity changes 12 hr after the dietary change. One way this can be demonstrated is by monitoring elevated DILP2 neuron activity throughout the 24 hr starvation or sucrose-only period using an transcriptional reporter of intracellular Ca^{2+} (e.g., TRIC reagent described by Gao et al., 2015). One would predict increased reporter activity in the IPCs following 24 hr starvation or sugar-only conditions.
10. In the Introduction, the authors write, "Flies potently suppress their sleep when starved, and at least some evidence suggests they are resilient to this form of sleep loss", referencing Keene et al., 2010, Thimgan et al., 2010, and Donlea et al., 2012. Additionally, in the Discussion section, the authors write, "It has been reported that starvation does not induce a sleep rebound", referencing Thimgan et al., 2010. In both the Thimgan et al. and Donlea et al. articles, the starvation-induced sleep deprivation employed was 12 hr in duration, not the 24 hr used in this manuscript. Furthermore, the claims made by this manuscript relies on an increased arousal threshold response happening at least 12 hr after starvation onset (Figure 2D,E,I,J), since it is inferred that this increased sleep depth helps compensate for the subsequent loss in recovery sleep. I question whether it is fair to compare the results of these papers with what the manuscript presents and suggest that these sentences be edited to address this concern.
