# Supplementary material for: Drosophila insulin-like peptide 2 mediates dietary regulation of sleep intensity
Source: PLoS Genet. 2020 Mar 11;16(3):e1008270. doi: 10.1371/journal.pgen.1008270 (PMC7089559; doi:10.1371/journal.pgen.1008270)
Supplement: Attachment — Submitted filename: Response to Reviewer Comments_Final.pdf [file pgen.1008270.s010.pdf]

Response for Reviewers:

All three reviewers and I agree that this manuscript addresses interesting questions about how different types of sleep loss influence sleep homeostasis and about how different nutrients modulate such relationships. In my opinion, the scope and subject are appropriate for the readers of PLOS Genetics. However, reviewers 1 and 3 list several major concerns that must be addressed before the manuscript can be considered further for publication. Some of these will require new data and significant revision to the manuscript structure. These include but are not limited to: (i) changes in how sleep and sleep rebound are measured and presented to ensure an appropriate time frame and (ii) additional evidence and mechanistic insight into role of Dilp2 signaling in modulating sleep intensity and rebound following starvation. If the authors believe that they can address these concerns in full, I would recommend a significant revision and resubmission, at which point I would ask the reviewers to evaluate the revised manuscript.

- We appreciate the careful reading and thoughtful comments from all three reviewers. We have significantly revised the manuscript through additional experiments, clarification of text, and substantial changes in data analysis and presentation. We believe it is now a much stronger paper and we are grateful for the feedback we have received.

Reviewer #1 Comments:

In this work by Brown and colleagues, the authors examine how distinct types of sleep loss affect sleep homeostasis. They focus on the idea that starvation-induced sleep loss does not result in subsequent sleep rebound because it is accompanied by deeper sleep during the period of starvation. They also begin to examine the mechanisms controlling these processes. The manuscript is interesting but could have gone further with regard to how Dilps connect to other sleep-wake systems known to affect both metabolism and sleep homeostasis. I had some major concerns about the manuscript and data as well.

- Thank you for these thoughts. We address the specific comments below.

Overall, the manuscript is not easy to follow. The authors should try to clarify the rationale of each new set of data and work harder to connect them with each other (connecting paragraphs and sections). Also, a majority of the data is in Supplemental figures, much of which seems as important as the main figures. It would help if more of the data is presented as main figures rather than supplemental.

- We have significantly restructured the manuscript. We have moved additional figures into the main text and worked to present all figures as clearly as possible.

Major points:
1. A major critique is that sleep rebound is measured over 12 hours of the recovery day. Work from many labs makes clear that changes might be washed out over this time frame. Given this importance, measurements of sleep duration, rebound, and depth should be broken down into bins, showing at a minimum whether the absence of change with starvation holds when looking at hours 0-1 of recovery, 0-3, and 0-6. That can all be incorporated to make Fig 1 a more in depth report of the data (rather than only show min/hour as averaged over all 12 hours). This should include sleep traces.

- We fully agree with this suggestion. In the revised version, we have added profiles of sleep duration and arousal threshold throughout the manuscript. In addition, we have also included measurements of homeostatic rebound that have been binned into 0-3, 0-6, and 0-12 hours of recovery.

2. Why is sleep loss only measured over the night if 24 hrs of sleep restriction was used? And why was 24 hrs of sleep deprivation chosen rather than 12 hrs of night only, given that day and night sleep are likely different based on many lines of evidence? If day sleep loss with each manipulation is different, this could easily be a driver of differences reported regarding sleep depth at night, and next day recovery. As in point 1, this data could be shown in sleep traces.

- Our rationale for measuring sleep duration and arousal threshold only at night was based on our findings that the effects of starvation on these traits occurs only after 12 hrs have passed. Our rationale is similar for why we choose 24 hrs of sleep deprivation instead of 12 hrs. We now include sleep traces as well as daytime and nighttime data in all figures, as appropriate. The notion that 12 hours of starvation is required to induce changes in sleep intensity, rather than it being a nighttime specific effect is further supported by data where we starved flies for 36 hrs (now Figure 3).

3. The LL data (Fig 1D) is flawed regarding rebound. These flies are going to be arrhythmic so will redistribute sleep between night and day, which then will appear to show an increase in sleep over 12 hours of day. However, this is not necessarily rebound sleep, and instead might reflect a change in when sleep occurs from a circadian perspective.

- We thank the reviewer for these comments. We agree that there are a number of confounds with this data set. Our initial purpose for including these data is that the finding that sleep loss via constant light induces a rebound the following day is in agreement with other forms of sleep deprivation used (other than starvation). We have conferred with two leading circadian labs and they also agree with the reviewer that circadian and sleep homeostat effects are difficult to separate experimentally. We have not been able to come up with a practical way to use light to induce deprivation without this confound. For this reason, we have removed the constant lighting experimental treatment. It does not change the overall conclusions of the manuscript.

4. The authors show that length of starvation drives changes to sleep depth. How does length of starvation affect sleep rebound duration? After 36 hours of starvation, does sleep rebound now emerge? 48 hrs? This depends on how long they can be starved, of course. My main question (related also to point 1) is whether starved flies ever show sleep rebound.

- We find that 36 hrs of starvation also does not produce a homeostatic rebound. We have added these results as Figure 3 in the main text. After 48 hrs of starvation, we observe an approximately ~30% drop in survival and so we did not include these data. Regarding these experiments, we now state in the Results section: "To determine whether longer periods of starvation induce recovery sleep, we extended starvation for 36 hours beginning at ZT12 (onset of lights off; Fig 3A), resulting in over 500 minutes of total sleep loss (Fig 3B,C). Arousal threshold was elevated from hours 12-24 and 24-46 following starvation, suggesting sleep depth is increased, even under severe starvation conditions (Fig 3D,E). Despite the robust loss in sleep, change in sleep duration or arousal threshold was detected when flies were returned to food

(Fig 3F,G). Therefore, flies do not exhibit a homeostatic rebound following prolonged periods of starvation, even though it results in significant cumulative sleep loss." (line 148).

5. The authors report increased night sleep depth on sucrose compared to other foods (Fig 3C). Does sucrose also result in increased sleep depth across the 24 hour day, or only during night? Would be interesting to know whether the day/night sleep depth (which can depend a lot on background according to published work) is more or less robust depending on the food.

- We find that a sucrose-only diet increases arousal threshold only at night. To show this, we have added measurements of sleep depth during the day to this figure (now Figure 4E).

6. What happens to sleep duration during recovery in schematic of Fig 3A? Even though sleep duration is not changed on sucrose (no sleep loss), those flies sleep more deeply. Does this affect subsequent next day sleep in some way?

- We observe no change in sleep duration or arousal threshold during the recovery period following 24 hrs of a sucrose-only diet. We have added these findings as an additional panel in this figure (now Figure 4F,G). We now state in the Results section: "Given that flies increase arousal threshold on a sucrose-only diet, we next assessed whether this would have any effect during recovery. We found no change in sleep duration or arousal threshold in the subsequent 12 hrs after sucrose-only feeding (Figure 4F,G). This suggests that even though flies fed a sucrose-only diet sleep more deeply, there is no effect on sleep duration or depth during later time periods" (line 165).

7. The 2DG experiments omit a critical part in the comparison to starved flies: do they exhibit rebound from a sleep duration perspective? If so, then it calls into question whether this phenocopies starved flies. In general, the 2DG line of logic is not presented clearly. The authors need to add another 1-2 lines of rational to explain how this addresses the contribution of sensory processing to sleep/arousal. I was not able to follow the concluding sentence of this paragraph for this reason. As it stands, this result (Fig S5) seems like a distraction. Moreover, this seems like an indirect way to ask whether the sleep depth changes emerge from sensory differences (tasting certain foods drives different sleep) as opposed to metabolic changes. Can the authors show more directly that sleep changes are related to sensory differences?

- We agree that the rational for this experiment was not clearly presented, and we now provide additional data, as well as descriptions to justify these experiments. We observe no change in sleep duration or arousal threshold during recovery following 24 hrs of 2DG feeding, regardless of rebound duration. We have added these findings as an additional panel in this figure (now Figure 5F,G). We also no longer discuss sensory processing and instead introduce the use of 2DG as a way to pharmacologically induce metabolic deprivation. We have moved experiments using 2DG to the main text and have clarified our rationale for performing these experiments. We now state: "To determine whether the effects of starvation can be recapitulated by inhibition glycolysis, and thereby preventing cellular utilization of sugars and numerous dietary amino acids, we pharmacologically starved flies by feeding them standard fly food laced with the glycolysis inhibitor 2-deoxyglucose (2DG; Fig 5A; [38,39]). In agreement with our previous findings [39], flies fed 2DG slept less than those housed on standard food (Fig 5B,C). Further, this decrease in sleep duration was accompanied by an increase in arousal threshold (Fig 5D,E), largely phenocopying starved flies. Further, there was no rebound in sleep duration or arousal

threshold when 2DG-treated flies were placed back on standard food (Fig 5F,G), suggesting the elevated arousal threshold induced by 2DG is protective against sleep debt. Together, these findings suggest that the changes in sleep duration and arousal threshold in starved flies is a result of metabolic deprivation." (line 176).

8. As with the point above, I do not understand the contribution of Fig S6. Here the authors show that sleep in the SAMM is the same as sleep measured in Fig S5, and that metabolic inhibition with 2DG inhibits metabolism as measured in the SAMM. Is this all just proof of the SAMM system working basically?

- Indeed, our results that sleep is also suppressed in the SAMM system is to demonstrate that we observe similar effects in both assay systems. It is important to note that the SAMM system is a new system (we have developed and published in two previous manuscripts) and it is fundamentally different from the DAM system that is classically used. For example, there is air flowing through the chamber in the SAMM, there are three IR beams, and the arena is significantly larger. However, so as to minimize confusion, we have decided to remove all experiments using the SAMM system from the manuscript.

9. For Dilp2 RNAi, what about day sleep duration? Is this changed, which could affect sleep depth at night?

- In agreement with our findings using w^{1118} flies, we find that daytime sleep duration is also reduced in the $Dilp2^{RNAi}$ flies. We have now added daytime sleep and arousal threshold measurements to this figure (now Figure 6).

10. Also, For Dilp2 RNAi, is it correct that the lack of deeper sleep during recovery (Fig 4G) is unexpected, compared to other forms of sleep loss that induce more, deep sleep (Fig 1C-E)? Can the authors comment on this? Same question for Fig 4N. With caff or LL (Fig S7), they now show increased sleep duration and depth during recovery. Why?

- We find that *Dilp2* flies do not increase their sleep depth during starvation, and as a likely consequence they require a rebound from starvation-induced sleep suppression. However, similar to other types of deprivation that induce a rebound (e.g. caffeine feeding), *Dilp2* flies increase arousal threshold during recovery sleep. We now present these data using $Dilp2^{RNAi}$ (Figure S5A-D) and $Dilp2^{null}$ (Figure S5E-H). Further, we do not observe a significant increase in arousal threshold using adult specific knockdown (*Dilp2*-GS), but data trends towards an increase (Figure S6B).

11. Overall, the authors, who were involved with previous work on how different forms of sleep loss affect sleep rebound, missed an opportunity to integrate OA, Dilps, metabolism, and sleep rebound. OA neurons feed into Dilps to affect sleep. Why not examine this in more depth by looking at how OA-based sleep loss (Tdc2>TrpA1) affects recovery arousal, arousal during sleep loss, interactions between different types of food, and metabolic rate, etc? Do Tdc2>TrpA1 flies at elevated temps (at which some sleep persists, ie not total sleep deprivation) show deeper sleep like starved flies, explaining why there is not subsequent rebound? With this manipulation, according to work this group contributed to, flies show persistent memory impairments, as opposed to starved flies. The authors should at least test whether Dilp2 manipulations during starvation (no increased sleep depth) DO show a subsequent memory deficit. And, then the authors can discuss how OA, Dilp2, and other

systems are potentially coupled (or dissociable).

- We agree with this assessment and have added discussion of possible interactions with octopamine neurons. We agree that it would be very interesting to test the effects of sleep loss on memory, as an additional feature of resilience. The experiments from our previous collaborative manuscript (Seidner *et al*., 2015) were technically challenging and required extensive protocol optimization. This is in part because the memory assay we used (aversive taste memory) requires starvation, and this (or the motivational component of the assay) impacted by *Dilp2* deficiency. We are certainly eager to initiate these experiments, but hope they can be part of future publications. In addition, a confound of linking the approaches in Seidner 2015 to this manuscript is that the former paper employed thermogenetic activation to OA neurons to induce sleep loss. While these neurons may be upstream of Dilp2 neurons, there are likely broad differences in brain function compared to starve flies. We now state in the Discussion: "*Dilp2*-expressing neurons are functionally downstream of wake-promoting octopamine neurons [14]. Induction of sleep loss by activating octopamine neurons does not induce a rebound [9], phenocopying loss of *Dilp2*. Together, these findings suggest a complex role for *Dilp2* and the IPCs in sleep regulation, and suggest multiple transmitters expressed in the IPCs may act in concert with *Dlip2* to differentially regulate sleep under fed and starved conditions. The presence of a rebound in Dilp2 mutants following starvation raises the possibility that these animals are experiencing sleep debt.

Minor:

12. Use of word "remarkably" in Abstract seems like an overstatement

- We agree that 'Remarkably' is not a particularly good word to use in science writing, and that it is an overstatement in this case. We have removed it from the abstract.

13. In Summary, "surprisingly little is known" also seems like an overstatement given that we now know a decent amount regarding recovery sleep mechanisms.

- We have changed this to "Despite the robustness of this feature, the neural mechanisms regulating recovery from different types of sleep deprivation are not fully understood" (line 36).

14. Lines 72-73: there must be a typo. Did the authors mean "…how different genetic…manipulations *that* differentially modulate sleep impact quality and homeostasis."? or "…how different genetic…manipulations differentially modulate sleep quality and homeostasis."

- Thank you for bringing this to our attention. The sentence now reads "However, a central question is how different genetic, pharmacological, and environmental manipulations impact sleep quality, and homeostasis." (line 65).

15. Fig S1C is referred to in the Results section out of order (after Fig S2), which caused a great deal of confusion while reading. Please move this to Fig S2 or on its own. Also, is the y axis labeled incorrectly? Should this be arousal? Is this different for starvation data than Fig 2E, or replotting the same data?

- We moved these data so that it is now included as part of a main figure (Figure 2G-J) and have correctly labeled the axes.

16. What is "NS" in Fig S2E measuring? Is there no significant difference in arousal in day vs night on any day?

- Indeed, we observe a significant difference in sleep duration between day and night, but no difference in sleep was observed over time (both during the day and during the night). To avoid confusion, we have removed the words NS and state this clearly in the figure legend.

17. Line 145: authors say "nighttime specific increase in sleep depth" immediately after presenting in CS flies that shows increase in sleep depth across day and night. Consider rephrasing to "increase in sleep depth during the night…is due to either…".

- We have made the appropriate change.

18. Is it correct that sleep duration measures in the SAMM system with 2DG show the same result as Fig S5? If so, the authors should just directly state that they get the same result in both, then move to the metabolic measures.

- We have removed all data using the SAMM system from the manuscript.

19. For schematic in 3A, do not show the recovery period since this is not examined.

- We have now added data on homeostatic rebound (after sucrose feeding) to this figure (now Figure 4) and so we have chosen to keep this part of the schematic.

20. In Fig 4I, what is "w1118>dilp2null"? H, I should match K.

- We have now made sure that all genotypes are consistently labeled.

21. Line 229: no rebound in sleep duration or depth after what manipulation? Sucrose feeding? If so, the control data needs to come when sucrose data is first presented. If something different, the authors need to clarify in the text, as this is difficult to follow. Fig legend (line 883) says the manipulation on sucrose "does not change daytime sleep duration". After moving back to regular food? Hard to know what is meant here.

- Correct, we observe no rebound in the 12 hrs after sucrose feeding (when flies are flipped back to standard food). We have re-worked this paragraph to be clearer. We now state: "Additionally, we did not observe any homeostatic rebound in sleep duration or depth in the control or in *Dilp2*-GAL4>UAS-*Dilp2*^{RNAi} flies (S7C and S7D Fig), suggesting that a homeostatic rebound, similar to what we observe in these flies after 24 hrs of starvation, occurs only after a decrease in both sleep duration and depth. We found similar results in *Dilp2*^{null} flies (S7E-H Fig). Overall, these results suggest that *Dilp2* uniquely regulates sleep depth both during starvation, and the absence of yeast." (line 235).

22. Lines 262-263: cellular basis of the homeostat is poorly understood. This is a surprising statement given many in depth papers on dFB and EB recently, at the molecular and cellular level.

- We agree. We meant to state that little is known about how the homeostat functions in response to different types of sleep loss. It has been revised to state “While the cellular basis homeostatic regulation in response to different types of sleep loss remains poorly understood.”

23. Line 282: The SNAP system is different than mechanical shaking using the vortexer, which is what most labs use. Judging from methods sections of fly sleep papers, few use the SNAP system.

- We have corrected the sentence to state: “In fruit flies, the vast majority of studies use mechanical shaking to induce sleep deprivation in flies [5,61–63].” (line 272).

24. Line 116 concludes that arousal changes are not the result of "circadian regulation", but the premise of the experiment is to show that changes are not from habituation to the stimulus. The conclusion is really the habituation point, not anything about circadian regulation.

- We have made the appropriate change.

25. Lines 232-234 end with "the resulting homeostatic rebound is independent of sleep depth". This is very confusing - my understanding of the manuscript is the whole point is that whether or not homeostatic rebound occurs depends on prior sleep depth.

- To avoid confusion, we have removed this statement from the text.

26. Line 242: the authors say "no difference in sleep" in dilp null flies, but what they mean I believe is "no difference in night sleep duration", since in fact they do show a difference in sleep depth in these flies compared to controls using other assays.

- We have made the appropriate changes.

28. Based on Fig 3, how can the authors conclude that loss of protein per se drives increased sleep depth, as opposed to any diet that decreases metabolic rate also increasing depth? The Dilp experiments do not dissociate this because Dilps seem to be required for dietary changes to alter sleep depth. I would particularly consider changing how strongly this is stated in line 92 ("critical role").

- We have removed the word critical.

Reviewer #2 Comments:

This is a very interesting and well written paper on the specific role of dilp2 in sleep regulation. It makes a significant contribution to our understanding of the interaction between diet, metabolism and sleep. I just have a few suggestions for minor corrections.

- We appreciate your input and hope we have sufficiently addressed your comments below.

1. Check the references - eg. reference #73 has the wrong author list.

- We have double checked the authors and formatting for all references.

2. Figures - the figures are a little confusing and would benefit from clearer labelling and legends. For example, in figure 4, it is difficult to determine which panels represent day vs night sleep and it is unclear what is the difference between panels D,E and F,G.

- In an effort to make sure our data can be clearly interpreted, we now include a schematic of our experimental design and a color-coded legend in all main figures. We also include sleep profiles throughout the manuscript, which are likely easier to interpret.

3. Genetic background of fly stocks - although the authors state that they use 2 different backgrounds (w1118 and CS) it is not clear if the GAL4/UAS stocks have been backcrossed to the appropriate genetic background. Please clarify.

- Indeed, all lines have been backcrossed to the w^{1118} fly strain. We have clarified this to the methods. We state: "The *Dilp2*^{null} flies as well as all GAL4 and UAS lines were backcrossed to the w^{1118} laboratory strain for 6 generations. The Canton-S strain was used to validate that the observed effects were not specific to the w^{1118} strain (S3E-H Fig)." (line 352).

Reviewer #3 Comments:

Overall, the findings and conclusions drawn are noteworthy and novel. However, I have several general issues with this manuscript that I believe must be addressed, including (i) possible inconsistencies between the starvation-induced sleep intensity and rebound phenotypes reported here and published previously by others; (ii) the lack of pursuit to identify the macronutrient responsible for the changes in sleep depth; and (iii) the minimal evidence supporting a role for DILP2 signaling in modulating sleep intensity and rebound following starvation, along with its connection to dietary yeast. If the authors address the following concerns, that would greatly strengthen my opinion of the manuscript and could merit publication in PLOS Genetics.

- We appreciate your critical review of our manuscript and hope we have sufficiently addressed your comments below.

1. According to data presented in Figure 1, the authors conclude that starvation-induced sleep loss does not produce a subsequent increase in rebound sleep. However, Keene et al. previously reported that "male and female flies rebound in the 4 hr after food deprivation" (Keene et al., 2010), contradicting a major conclusion made in this manuscript. Can the authors comment on or resolve this discrepancy?

- We apologize for this confusion. In the initial paper, we indeed saw a rebound after four hours, and this conflicts with the findings in this paper. There are a number of aspects about the way we performed that experiment that are (regrettably) not satisfactory. First, the experiment was performed in single-beam DAMS (rather than video tracking) and what we initially interpreted as rebound may have been the flies feeding during recovery. Second, measurement of sleep were likely less precise than in the video-tracking used here. We did visually observe flies (as stated in the paper), but clearly this was not quantitative. I wish I had thought of these factors when performing the experiments, but the field's understanding of sleep, along with the tools available for measuring sleep (such as multi-beam DAMS and the DART system) have significantly improved over the past 10 years. In fact, a large impetus for this work was to

systematically compare different types of deprivation to, at least in part, address different results throughout the literature. While we are more than happy to include this discussion within the manuscript, we worry that it would be a distraction. We feel the best way of addressing these differences is with the careful analysis presented in this manuscript.

2. Regarding Figure 1, it is worth noting that the greatest differences in recovery sleep are observed within the first few hours immediately following an acute sleep deprivation protocol (Shaw et al., 2000; Hendricks et al., 2000; Huber et al., 2004; Keene et al., 2010; Dubowy et al., 2016; Vienne et al., 2016; Sonn et al., 2018). Since the authors quantified recovery sleep across a 12 hr interval, immediate changes in recovery sleep may have gone unnoticed. I recommend presenting sleep amount profiles illustrating the baseline, sleep deprivation, and post-deprivation recovery period alongside the 12 hr recovery violin plots. This would allow the authors to further demonstrate how sleep rebound does/does not change across time for each of the different sleep deprivation paradigms presented in Figure 1.

- We fully agree and have added profiles of sleep duration and arousal threshold to the figures as appropriate. We have also included measurements of homeostatic rebound that have been binned into 0-3, 0-6, and 0-12 hours of recovery.

3. Have the authors looked at sleep fragmentation and architecture parameters during either the sleep deprivation period or 24 hr recovery period in an attempt to independently support the arousal threshold observations? For example, one would expect an increase in average sleep bout duration and a decrease in the number of sleep bouts to complement an increase in sleep depth; a trend that would be observed in 24 hr starved flies during the night period. Such an analysis would have to be done without the use of the DART system, since hourly disruption of sleep could alter interpretation of the fragmentation parameters.

- We thank the reviewer for the suggestion. We have now included data using the *Drosophila* activity monitoring system (now S2 Fig). We now assess how sleep architecture changes as a result of the difference methods of sleep deprivation used in our study. Our findings are described below, which have been added to the results section of the manuscript. We state: "To assess how sleep architecture is affected by these methods of sleep deprivation, we measured sleep using *Drosophila* activity monitors (DAM; [27]). Similar to our results obtained in the DART, we found that sleep is significantly reduced when flies are starved, mechanically shaken, and when fed caffeine. When flies are fed caffeine, the number of bouts remain unchanged, but their length significantly decreases (S2A-E Fig). When flies are mechanically sleep deprived, both bout number and length are significantly reduced (S2F-H Fig). Lastly, starvation-induced sleep suppression results from a significant decrease in bout number, but not bout length (S2I-K Fig). Given that deeper sleep states are correlated with longer sleep bouts [24,25,28], these findings provide additional support for our findings that sleep depth increases during starvation." (line 121).

4. The Materials and Methods section describes how arousal threshold was tested, yet never explains how this data was used to define and quantify arousal threshold throughout the manuscript. Exactly what value is being reported in the arousal threshold plots? Does it refer to the average amount of stimulus required to arouse a specified percentage of the sleeping population?

- The value being reported is the proportion of the maximum force applied to the platform, thus an arousal threshold of 0.4 is 40% the force of 1.2g. To clarify, we have changed these values to percentages in all the figures. We have also modified our statement in the methods section. We now state: "Measurements of arousal threshold are reported as the proportion of the maximum force applied to the platform, thus an arousal threshold of 40% g is 40% of 1.2g." (line 374).

5. Even though the authors conclude that changes to sleep intensity and metabolic rate are primarily driven by dietary yeast availability, little is said or done to determine which macronutrient provided by dietary yeast is responsible for these changes. The evidence strongly points towards a role for protein and/or amino acids in modulating sleep depth, which would be consistent with recent findings demonstrating the effects that specific amino acids have on sleep regulatory processes (Dai et al., 2019; Sonn et al., 2018; Ki and Lim, 2019). Could the authors (i) use a chemically defined food recipe devoid of carboyhydrates (e.g., Lee and Micchelli, 2013) in place of dietary yeast alone conditions, with the expectation that it phenocopies the dietary yeast alone response or (ii) by systematically adding back specific amino acids to a sugar-only diet in an attempt to revert the sleep intensity phenotype?

- Per the reviewer's recommendation, we have added amino acids to sucrose and then measured sleep and arousal threshold. The amino acids comprise both essential and nonessential amino acids, and were administered as previously described (Liu *et al*., 2017). We find that upon the addition of amino acids, sleep depth is restored to levels similar as that of standard food and yeast food treatments. We have added these data to the manuscript (Figure 4H-K). We now state in the results section: "To specifically examine the contribution of amino acids to the regulation of arousal threshold, we supplemented sugar with a cocktail of amino acids using a previously described protocol [37]. In agreement with previous findings, sleep duration did not differ in flies fed standard food, sucrose, or sucrose and amino acids (Fig 4H,I). The addition of amino acids to a sucrose only diet restored nighttime arousal threshold to the level of flies fed standard food (Fig 4J,K). These findings suggest the absence of dietary amino acids increases sleep depth without affecting sleep duration." (line 169).

6. Flies fed a standard yeast-sugar diet containing the glycolysis inhibitor 2-deoxygluose caused a decrease in sleep duration and increase in arousal threshold, which is used to establish that "metabolic deprivation, rather than lack of sensory inputs, account for the changes in sleep…" (Figure S5). These results also imply that glucose metabolism is capable of modulating sleep intensity, despite the observation that environmental sugar availability does not drive changes in sleep depth. Can the authors comment on or resolve this possible conflict? Furthermore, could experiments be designed to assess the contribution of protein metabolism/homeostasis to changes in arousal threshold 12 hr after dietary change onset, which would better complement the data implicating the effects of dietary yeast on sleep intensity?

- As described in response to Reviewer 1, we have now modified the justification for the 2DG experiment and also include analysis of rebound following 2DG treatment. We clarify that 2DG generally disrupts glycolysis impacting utilization of sugars and many dietary amino acids. The text now states: "To determine whether the effects of starvation can be recapitulated by inhibition glycolysis, and thereby preventing cellular utilization of sugars and numerous dietary amino acids, we pharmacologically starved flies by feeding them standard fly food laced with the glycolysis inhibitor 2-deoxyglucose (2DG; Fig 5A; [38,39])." (line 176).

7. Given the widespread physiological behavioral changes that often accompany DILP2 loss throughout development, have the authors considered assessing whether acute loss of DILP2 in adults flies during the starvation period is enough to suppress sleep depth during starvation and promote sleep rebound post-starvation? The authors can either try (i) *DILP2-GeneSwitch-GAL4* driving *UAS-DILP2-RNAi* or (ii) [*DILP2-GAL4 + tubGAL80ts*] driving *UAS-DILP2-RNAi* to address this question.

- We thank the reviewer for the suggestion. We find that selective loss of *Dilp2* during adulthood is sufficient to suppress arousal threshold during starvation as well as produce a sleep rebound in the 12 hrs following starvation. We have added the following to the results section: "It is possible that *Dilp2* functions acutely to increase sleep depth during starvation, or that it is required during development. To differentiate between these possibilities, we utilized *Dilp2*-Geneswithch (GS) to temporally silence *Dilp2* expression in adulthood [47]. We acutely fed RU486 to flies harboring the transgene for inducible GAL4 in *Dilp2*-expressing neurons (*Dilp2*-GS-GAL4) as well as UAS-*Dilp2*^{RNAi} for 24 hrs prior to and during experimental manipulations (Fig 7A). We found that sleep duration in *Dilp2*-GS-GAL4>UAS-*Dilp2*^{RNAi} flies fed RU486 did not differ in the fed or starved state compared to its respective controls (Fig 7B; S6A Fig). Nighttime arousal threshold was significantly increased in all starved controls, but did not differ between fed and starved *Dilp2*-GS-GAL4>UAS-*Dilp2*^{RNAi} flies fed RU486 (Fig 7C,D), suggesting *Dilp2* is required in adults for starvation-dependent changes in arousal threshold. While control flies did not exhibit a rebound following deprivation, *Dilp2*-GS-GAL4>UAS-*Dilp2*^{RNAi} flies fed RU486 displayed a significant sleep rebound following starvation, suggesting *Dilp2* is required during adulthood for increased sleep depth during starvation that likely prevents rebound (Fig 7E). No effect on arousal threshold was observed during recovery in any of the manipulations (S6B Fig). Taken together, these findings suggest *Dilp2* acts acutely to increase sleep depth during starvation and confer resiliency to starvation-induced sleep deficits" (line 215).

8. Does acute overexpression of DILP2 in the IPCs (e.g., *DILP2-GeneSwitch-GAL4* driving *UAS-DILP*) cause an increase in sleep depth on standard diet or yeast-only? If it does, this experiment would further support DILP2 signaling involvement in modulating sleep depth.

- Indeed, we find that acute overexpression of *Dilp2* in *Dilp2*-expressing neurons increases arousal threshold in the fed state. Below we have shown the results in the fed state, and no differences were detected for any groups in the starved state. The only difference we observe is an increase in nighttime arousal threshold (B, Red with grey fill) with Dilp2 overexpression. While this experiment is generally in agreement with our findings, we thought it best to leave them out of the manuscript as the loss of function results have clearer interpretions. We are happy to include them if the reviewers or editors find they are important.

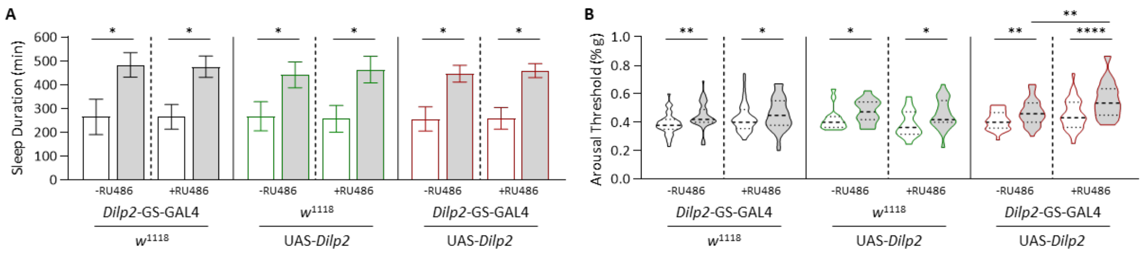

Fig 1. *Dilp2* overexpression acutely regulates arousal threshold in the fed state. (A) While all treatments increase sleep at night (grey bars) compared to day (white bars) (two-way ANOVA: $F_{1,298}$ = 43.11, *P*<0.0001), there is no effect of genotype (two-way ANOVA: $F_{5,298}$ = 0.05, *P*<0.99) or RU486 administration (two-way ANOVA: $F_{1,149}$ = 0.06, *P*<0.80) on nighttime sleep duration. (B) While all treatments increase arousal threshold at night (REML: $F_{1,176}$ = 53.01, *P*<0.0001), there is a significant interaction between genotype and RU486 administration on nighttime arousal threshold (REML: $F_{2,87}$ = 3.93, *P*<0.02). Post hoc analyses revealed that while nighttime arousal threshold remains unchanged among controls (*Dilp*2-GS-GAL4>w^{1118}: *P*<0.92; w^{1118}>UAS-*Dilp2*: *P*<0.86), overexpression of *Dilp2* in *Dilp2*-expressing neurons significantly increases arousal threshold (*Dilp*2-GS-GAL4>UAS-*Dilp2*: *P*<0.0018). For sleep measurements, error bars represent +/- standard error from the mean. For arousal threshold measurements, the median (dashed line) as well as 25th and 75th percentiles (dotted lines) are shown. * = *P*<0.05; [illegible]

9. Can the authors provide additional evidence that DILP2 signaling and/or DILP2 IPC activity is enhanced in response to their starvation or sucrose-only dietary conditions? While the Discussion section mentions "there are conflicting reports on whether *Dilp2* expression is modulated during starvation", I'm hoping the authors can provide clarity to this situation in this manuscript, which would further strengthen the case that increased DILP2 signaling is indeed responsible for the sleep intensity changes 12 hr after the dietary change. One way this can be demonstrated is by monitoring elevated DILP2 neuron activity throughout the 24 hr starvation or sucrose-only period using an transcriptional reporter of intracellular Ca2+ (e.g., TRIC reagent described by Gao et al., 2015). One would predict increased reporter activity in the IPCs following 24 hr starvation or sugar-only conditions.

- We agree that these are potentially exciting experiments. We are currently working to optimize these experiments using TRIC and CaLEXA, however we believe the optimization process will be time consuming and hope we can include these in a future manuscript. We have added the potential for these experiments to inform our understanding of IPC function to the discussion: "The IPCs have long been proposed as critical integrators of behavior and metabolic function [14,40,44,73]. Our results raise the possibility that *Dilp2*-expressing neurons become active on diets deficient in amino acids. The application of genetically encoded Ca^{2+} sensors that can be measured in freely moving animals, such as CaMPARI and TRIC, provide the opportunity to determine the effects of dietary macronutrients on IPC activity [74,75]." (line 303).

10. In the Introduction, the authors write, "Flies potently suppress their sleep when starved, and at least some evidence suggests they are resilient to this form of sleep loss", referencing Keene et al., 2010, Thimgan et al., 2010, and Donlea et al., 2012. Additionally, in the Discussion section, the authors write, "It has been reported that starvation does not induce a sleep rebound", referencing Thimgan et al., 2010. In both the Thimgan et al. and Donlea et al. articles, the starvation-induced sleep deprivation employed was 12 hr in duration, not the 24 hr used in this manuscript. Furthermore, the claims made by this manuscript relies on an increased arousal threshold response happening at least 12 hr after starvation onset (Figure 2D,E,I,J), since it is inferred that this increased sleep depth helps

compensate for the subsequent loss in recovery sleep. I question whether it is fair to compare the results of these papers with what the manuscript presents and suggest that these sentences be edited to address this concern.

- Thank you for bringing this to our attention. Again, we hope that dedicating an entire manuscript to starvation and rebound will be helpful to the field. We believe that as a general principle, our results are supported by literature suggesting flies are resilient to starvation-induced sleep loss. We have modified these sentences to be more precise. We state in the introduction: “Flies potently suppress their sleep when starved, and although the duration of starvation varies among studies, at least some evidence suggests they are resilient to this form of sleep loss [11,22,23].” (line 66). In the discussion we now state: “It has previously been reported that starvation does not induce a sleep rebound after 12 hrs of starvation [22], and in appetitive conditioning assays, starvation is required for memory formation, suggesting flies still form robust memories despite sleep loss [49–51]” (line 245).
